# Adult hippocampal neurogenesis poststroke: More new granule cells but aberrant morphology and impaired spatial memory

**DOI:** 10.1371/journal.pone.0183463

**Published:** 2017-09-14

**Authors:** Florus Woitke, Mihai Ceanga, Max Rudolph, Fanny Niv, Otto W. Witte, Christoph Redecker, Albrecht Kunze, Silke Keiner

**Affiliations:** Hans Berger Department of Neurology, Jena University Hospital, Am Klinikum 1, Jena, Germany; University of Lethbridge, CANADA

## Abstract

Stroke significantly stimulates neurogenesis in the adult dentate gyrus, though the functional role of this postlesional response is mostly unclear. Recent findings suggest that newborn neurons generated in the context of stroke may fail to correctly integrate into pre-existing networks. We hypothesized that increased neurogenesis in the dentate gyrus following stroke is associated with aberrant neurogenesis and impairment of hippocampus-dependent memory. To address these questions we used the middle cerebral artery occlusion model (MCAO) in mice. Animals were housed either under standard conditions or with free access to running wheels. Newborn granule cells were labelled with the thymidine analoque EdU and retroviral vectors. To assess memory performance, we employed a modified version of the Morris water maze (MWM) allowing differentiation between hippocampus dependent and independent learning strategies. Newborn neurons were morphologically analyzed using confocal microscopy and Neurolucida system at 7 weeks. We found that neurogenesis was significantly increased following MCAO. Animals with MCAO needed more time to localize the platform and employed less hippocampus-dependent search strategies in MWM versus controls. Confocal studies revealed an aberrant cell morphology with basal dendrites and an ectopic location (e.g. hilus) of new granule cells born in the ischemic brain. Running increased the number of new neurons but also enhanced aberrant neurogenesis. Running, did not improve the general performance in the MWM but slightly promoted the application of precise spatial search strategies. In conclusion, ischemic insults cause hippocampal-dependent memory deficits which are associated with aberrant neurogenesis in the dentate gyrus indicating ischemia-induced maladaptive plasticity in the hippocampus.

## Introduction

Ischemic brain insults are a leading cause of mortality and chronic disability in humans. In addition to causing a broad spectrum of physical symptoms, stroke increases the risk of dementia and brings about a steeper decrease in cognitive function in elderly people [1]. Recent studies support this concept and provide evidence that stroke impairs hippocampal function and accelerates age-associated memory decline in humans although the insults do not primarily affect the temporal lobe [2,3]. The cellular mechanisms of how ischemic infarcts cause alteration of hippocampal function are only partially understood.

The hippocampus is one of the neurogenic regions of the adult brain where neurons are continuously generated throughout life. The new granule cells (GC) are born in the subgranular zone of the dentate gyrus (DG), migrate in the granule cell layer, and become functionally integrated into neuronal networks within four to six weeks. The generation and integration seems to be strictly regulated and aberrant GC are extremely rare (< 1%) in the intact brain [4]. Analysis of the function of the newly born GCs has been challenging in the past but recent studies reveal that they are centrally involved in the formation and retrieval of spatial memories, particularly in the separation of complex visual patterns [5–7].

Ischemic insults stimulate adult neurogenesis in the DG [8]. However, the presence of more new neurons in the ischemic brain is not reliably associated with better outcome in memory tasks [9]. Recent findings even suggest that a significant portion of newborn neurons generated in the context of stroke reveal aberrant morphology and fail to correctly integrate into pre-existing networks [10]. Thus, stroke might rather negatively affect adult neurogenesis and subsequently impair spatial learning in mice.

In the present study, we asked the question of whether more new neurons are generated in the DG of mice with focal infarcts and whether they reveal specific hippocampus-dependent memory deficits in behavioural testing. And if so, whether there is evidence for aberrant hippocampal neurogenesis following stroke? To answer these questions, we employed a modified version of the Morris water maze that allows assessment of spatial memory and differentiation of hippocampus-dependent versus hippocampus-independent search strategies [11]. Notably, neurogenesis does not appear to be beneficial for hippocampal memory per se but rather contributes to highly specific functional aspects of spatial learning. Hence, the modified MWM includes specific features (complex visual landmarks, no curtain, goal reversal after day 4) which challenge the function of newly generated neurons [7,12,13].

Quantification and morphological analysis of new neurons was performed following distinct labeling techniques with retroviral vectors or with the thymidine analogue 5-ethynyl-2′-deoxyuridine (EdU). In a second set of experiments, we used running as a strong neurogenic stimulus and investigated whether this further enhances neurogenesis and possibly improves memory performance poststroke.

## Methods

### Animals and stroke model

The study was performed on a total number of 40 adult male C57BL/6J mice (11 to 15 weeks of age, 19–28 g). Animals were purchased from Charles River (Sulzfeld, Germany) and bred in our local facilities. Twenty-four animals received ischemic infarcts and 16 animals were sham operated. Infarcts were induced by using the middle cerebral artery occlusion model (MCAO). Briefly, mice were anesthetized with 2.5% isoflorane in a N_2_O:O_2_ (3:1) mixture. Through a middle neck incision the right common carotid artery (CCA), the external carotid artery (ECA) and the internal carotid artery (ICA) were carefully dissected from surrounding nerves and fascia. The left common and external carotid artery was occluded with a 7.0-polyfilament (Medicon eG, Tuttlingen, Germany) and subsequently an arteriotomy was performed in the common carotid artery. A 6.0-monofilament suture (Doccol cooperation, Sharon, MA) with a rounded tip was inserted into the common carotid artery and advanced through the internal carotid artery to the ostium to occlude the middle cerebral artery. After 45 minutes, the suture was removed, the wound was closed, and the mice were allowed to recover. During the MCA occlusion body temperature was maintained at physiological level using a heating pad. Sham animals underwent anesthesia and surgical procedure in a similar manner to the treatment group with exception of occlusion of the middle cerebral artery.

The study was carried out in strict accordance with the recommendations of the European Commission on the protection of animals used for scientific purposes. All experimental procedures were performed according to the ARRIVE guidelines [14] and approved by the local specific authority for regulating animal experimentation (Landesamt für Verbraucherschutz, Bad Langensalza, Thuringia, Germany, Permit Number 02-053/12). All surgeries were carried out under isoflurane anesthesia, and all efforts were made to minimize the suffering of animals. Postoperative care included monitoring animals twice daily. The application of analgesics was not necessary. No adverse events occurred during postoperative care.

### Retroviral vectors

To label newly generated granule neurons, we used the retroviral vectors CAG-green fluorescent protein (GFP) or CAG-red fluorescent protein (RFP)[10]. The viral vectors were developed from a murine moloney leukemia virus and were produced by cotransfecting HEK 293 T cells with the compound promotor CAG, the reporter Gens GFP or RFP, the CMV enhancer protein, the coating glycoprotein of rabies virus VSV-G and the posttranscriptional regulator element Woodchuck Hepatitis-Virus (WPRE). The final titer reached was approximately 1×10^7^colony-forming units/mL.

### Experimental design

Adult mice were randomly allocated to four experimental groups (S1 Fig). Mice either underwent MCAO in the left hemisphere or were sham operated at day 0. Following surgery they were either housed under standard conditions or had free access to a running wheel for 7 weeks: 1. MCAO and standard housing (MCAO-ST, n = 12). 2. MCAO sham surgery and standard housing, (Sham-ST, n = 8). 3. MCAO and running wheel (MCAO-RU, n = 12). 4. MCAO sham surgery and running wheel (Sham-RU, n = 8). Four days after stroke mice were anesthesized and stereotactically injected with 1 μl of CAG-GFP retrovirus into the dentate gyrus (coordinates from the bregma were, x = 3.1 mm; y = 1.5 mm; z = 4.0 mm). Additionally, mice received daily injections of thymidine analogue, 5-ethynyl-2′-deoxyuridine (EdU, 50 mg/kg) from days 3 to 15.

### Morris water maze (MWM)

At six weeks poststroke, mice were trained in the reference memory version of the Morris water maze task to locate a hidden escape platform in a circular pool (1.80 m diameter). Water was made opaque with a non-toxic white milk powder and kept at a temperature of 20–21°C. Each mouse underwent 6 trials per day for 5 consecutive days with an inter-trial interval (ITI) of 30 mins. Mice were released from one of four possible starting points and allowed to search for up to 120 s for the platform. The starting position remained constant each day. If mice did not find the platform within 120 s, they were guided to the platform and allowed to remain there for at least 15 s. On day 4, the platform was moved to the opposite northwestern quadrant (reversal) where it also remained on day 5. Probe trials lasting 60 s were performed on day 4 (before starting the reversal learning) and on day 5 (after finishing reversal learning) without a platform, respectively. Swim paths were recorded using VideoMod 2 (TSE Version 6.04; Rostock, Deutschland) and further analyzed using Matlab (The Mathworks, USA).

### Tissue preparation and immunocytochemistry

At day 46 animals were deeply anesthetized with isoflurane and perfused through the ascending aorta with 4% paraformaldehyde in phosphate buffer (0.15 mol/L, pH 7.4). Following perfusion brains were postfixed in paraformaldehyde and sliced into 40-μm sections. EdU staining was conducted using Click-iT™ EdU imaging kit (Invitrogen, Carlsbad, CA) according to a modified manufacturer’s protocol. After washing the slices were incubated for 1 hour using a Click-iT™ reaction cocktail containing Click-iT™ reaction buffer, CuSO4, Alexa Fluor® 555 Azide, and reaction buffer additive.

In addition, immunofluorescence standard methods were applied to double-, triple- or quadruple-label cells as previously described [15]. The following primary and secondary antibodies were used: goat anti-GFP antibody (1:500; Acris, Herford, Germany), rat anti-RFP antibody (1:500; Abcam, Cambridge, UK), mouse antineuronal nuclei antigen (1:500; Chemicon, Temecula, CA), Cy5 antimouse (1:500; Dianova, Hamburg, Germany), Alexa Fluor 488 antigoat (1:500; Invitrogen, Carlsbad, CA) and Rhodamine antirat (1:500; Dianova, Hamburg, Germany),. Additionally, 6-diamidin-2-phenylindoldihydrochlorid (DAPI, Sigma–AldrichChemie) was employed for nuclear staining in immunofluorescence.

Immunofluorescence stainings were analyzed by confocal laser scanning microscopy and peroxidase stained cells were evaluated by light microscopy.

### Quantification and statistical analysis

Basic analysis of MWM included quantification of latencies (time to find the platform), distance (path lengths) and time that animals spent in the quadrants. Further detailed analyses were undertaken based on the raw time-tagged *xy*-coordinates using Matlab, Version 2012b (The Mathworks, Ismaning, Germany). Search strategies were classified according to parameters and an algorithm previously described by Garthe et al.[11]. EdU imaging Kit stained sections were analysed by using confocal fluorescence microscopy (LSM 710, Carl Zeiss Jena, Germany) with an 40× objective. EdU+ cells were counted on every sixth section (240 μm intervals) of the complete ipsi- and contralateral dentate gyrus (DG) for subgranular zone (SGZ) and granule cell layer (GCL) The SGZ was defined as a two cell soma thin layer between the GCL and the hilus in line with previous reports (Jin et al., 2001; Walter et al., 2010). Colocalisation studies of multiply labeled fluorescence sections were performed by using confocal microscopy (LSM 710, Carl Zeiss Jena, Germany).

To quantify neurogenesis, the phenotypes of EdU+ cells of both hippocampi were analyzed on every sixth section (240 μm interval) from the complete rostro-caudal extension of the dentate gyrus according to their coexpression of EdU and NeuN. Total numbers of newly generated neurons were then determined by calculating the ratio of the percentage of EdU+/NeuN+ cells to the total number of immunofluorescence stained EdU+ cells in the dentate gyrus.

To analyze phenotype and dendritic complexity of the virus-labeled cells, we employed confocal microscopy, z-stack imaging and the semiautomatic Neurolucida system (MicroBrightfield, Colchester, VT) as previously reported [10]. Neurons with aberrant morphology were defined as: 1) bipolar cells in the granule layer of the DG with basal dendritic processes directed toward the hilus; and (2) ectopically positioned cells located either beneath the subgranular zone in the hilus or in the extension of the adjacent CA region.

**Fig 1:**
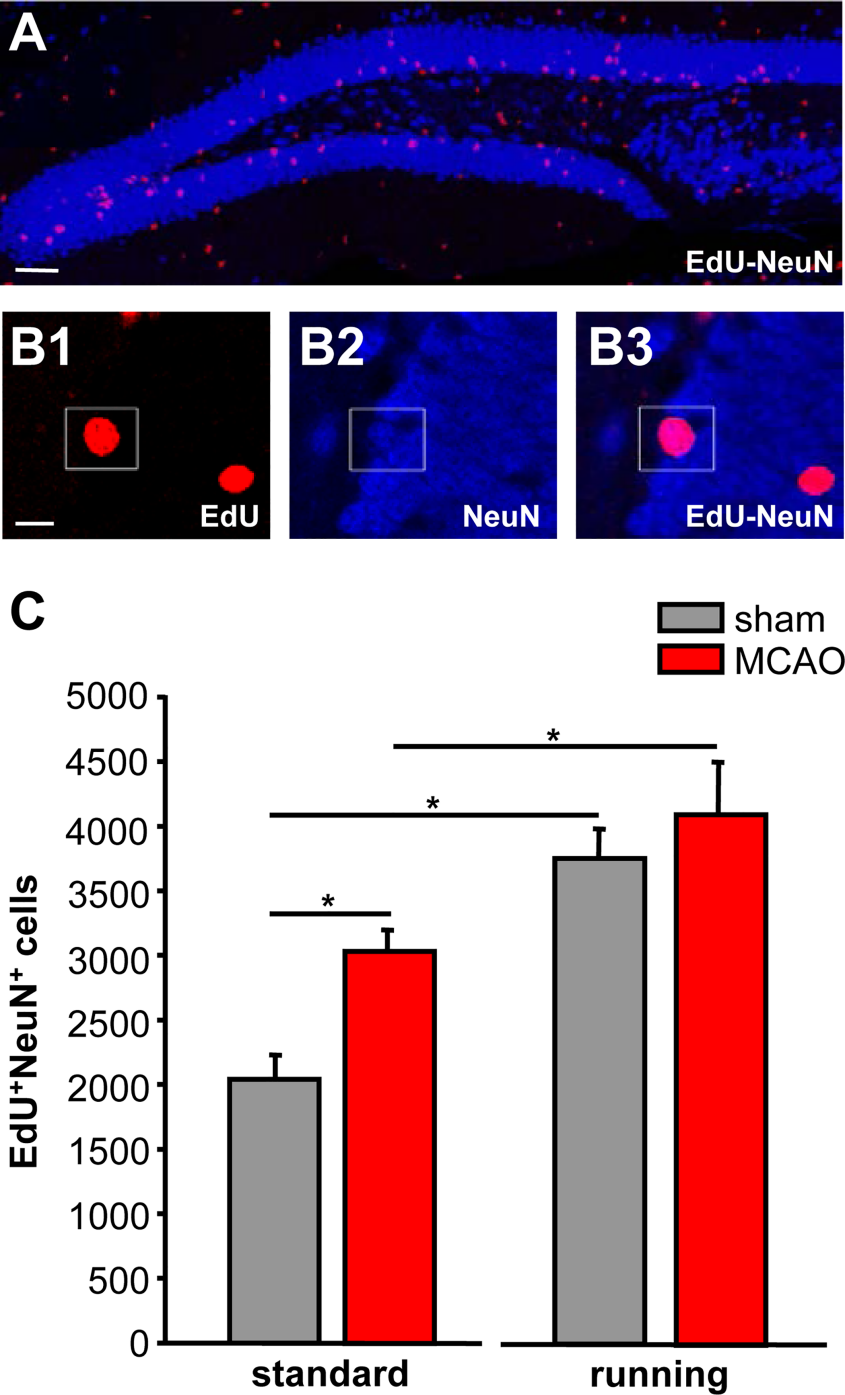
Stroke stimulates neurogenesis in adult dentate gyrus. **A,** Representative confocal image of dentate gyrus with newly generated neurons (red). B, Higher magnification of new neurons show colabeling of proliferation marker EdU (red) and mature neuronal marker NeuN (blue). **C,** Quantification of new neurons in animals housed under standard conditions or with free access to running wheels. Bars represent mean ± SEM. Asterisks indicate significant differences (p < 0.05). Scale bars represent 100 μm (A1), 10μm (B1).

**Fig 2:**
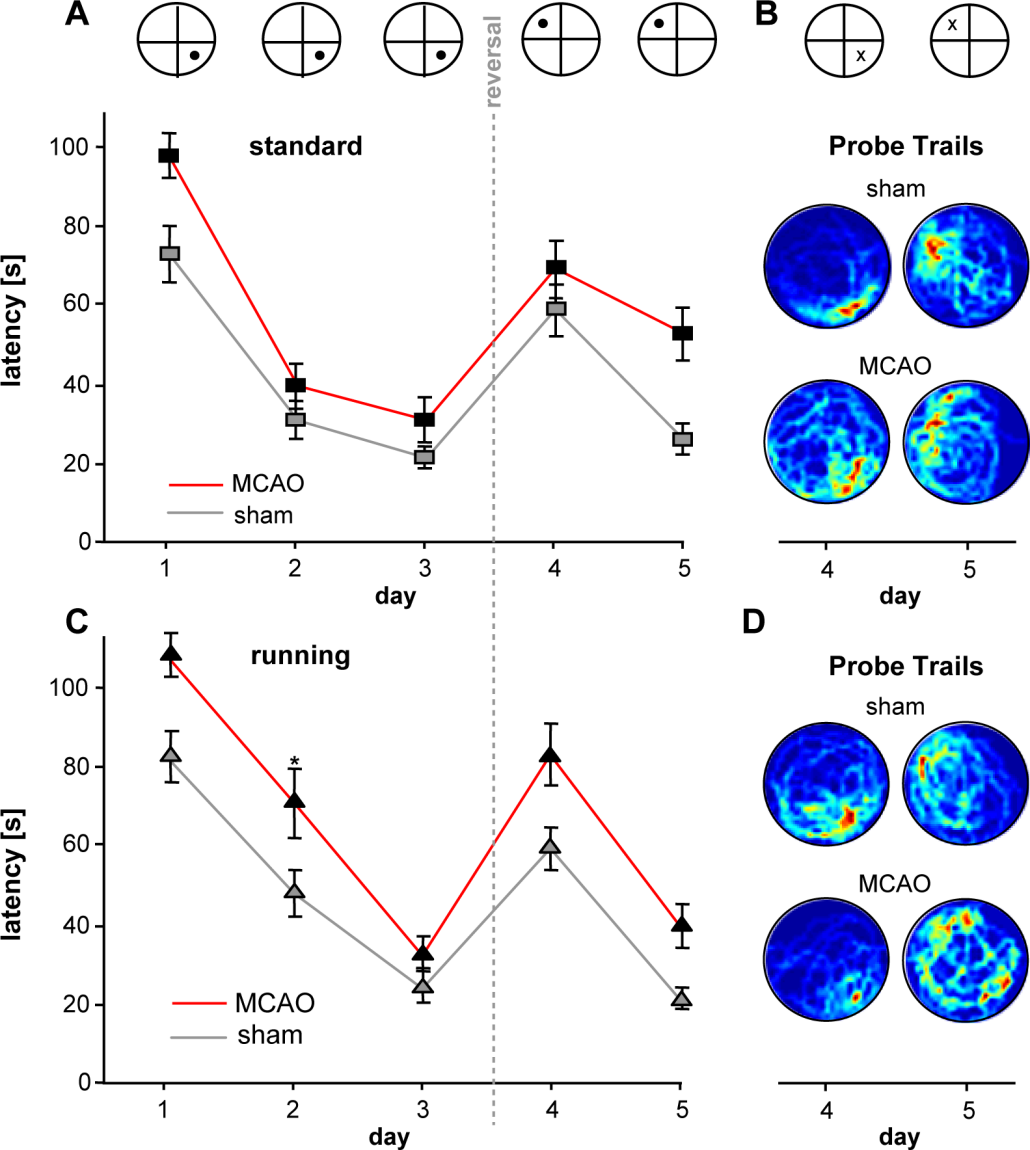
Spatial learning is impaired following stroke. **A/C,** Graphs reveal time (latencies) to navigate to the platform in MWM either following standard housing (A) or free access to running wheels (C), red = MCAO, grey = sham. **B/D** Heat maps of probe trails at days 4 and 5. Dark-red zones indicate a high presence probability. In the upper panel of A-B, the platform position is schematically illustrated. During the probe trails, the platform is removed and the putative position is marked with x.

**Fig 3:**
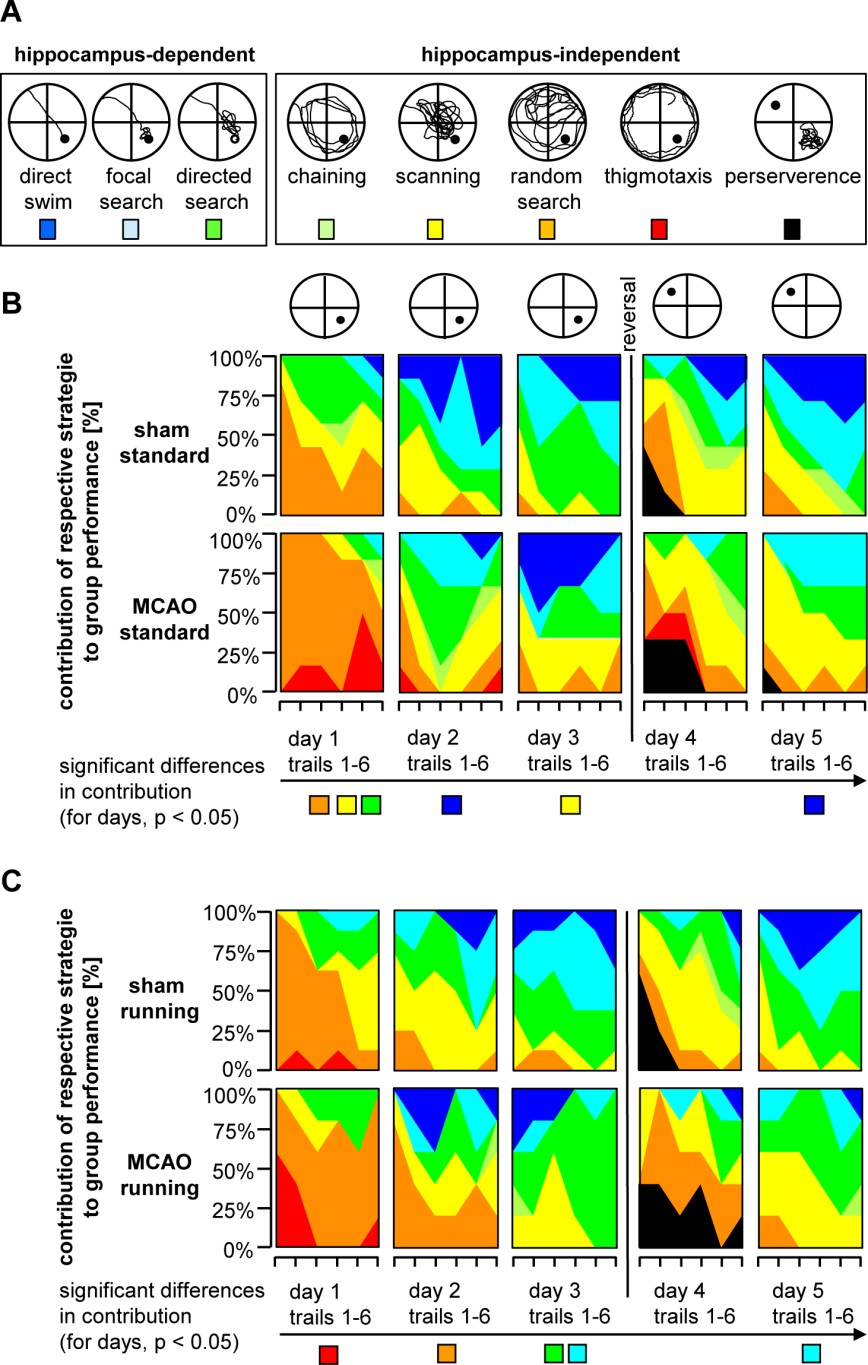
Stroke animals employ less hippocampus-dependent search strategies. **A**, Schematic illustration of distinct hippocampus-dependent and -independent search strategies in MWM. **B/C,** Contribution of single search strategies to group performance in MCAO- and sham operated animals either following standard housing (B) or free access to running wheels (C). Color code as indicated in A.

**Fig 4:**
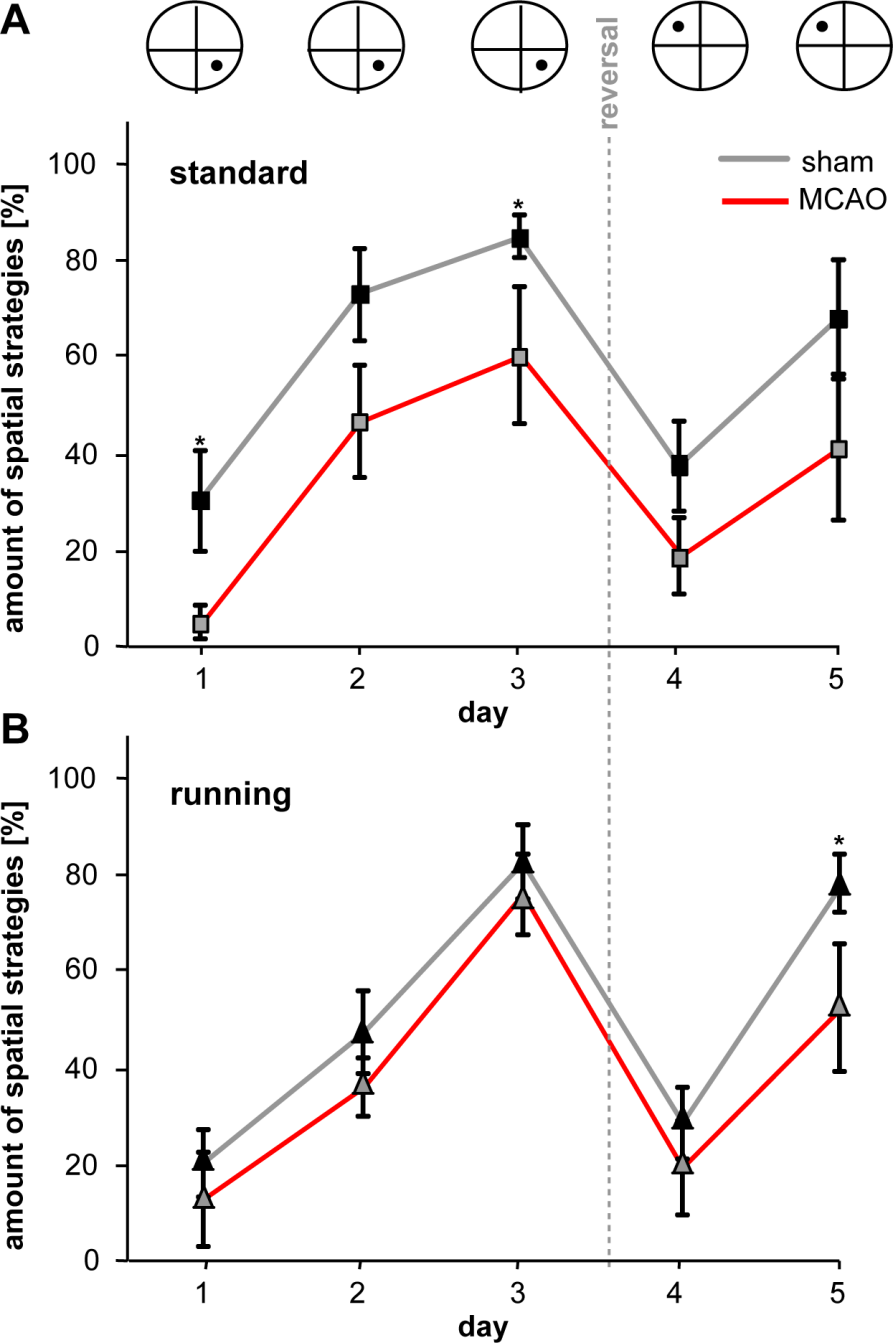
Contribution of pooled hippocampus-dependent search strategies. A/B, Graph depicts percentage of all hippocampus-dependent strategies (direct swim, focal search, directed search) in MCAO- and sham-operated animals either following standard housing (A) or free access to running wheels (B). Graphs represent mean ± SEM. Asterisks indicate significant differences (p < 0.05).

Statistical analyses were performed using SPSS 22.0 for Windows. Differences were assessed using one way analysis of variance (ANOVA) and post hoc Bonferroni correction for multiple testing (Comparison of cell numbers, Fig 1), ANOVA for repeated measurements (Comparisons of latencies and distance in MWM, Fig 2) and general equation estimation (GEE, comparsion of strategies in MWM, Figs 3 and 4) that allows estimation of odds ratios. Data are given as mean ± SEM unless otherwise noted. P values < 0.05 were considered statistically significant.

## Results

### Morphology of the ischemic infarcts

All animals analyzed in the MCAO subgroups showed typical focal infarcts covering the basal ganglia in the left middle cerebral artery territory (S1 Fig). The median infarct volume was 6.88 ± 3,40 mm^3^ (n = 11). It did not significantly differ between the MCAO-ST and MCAO-RU groups. Animals with extended ischemic damage affecting the hippocampus were excluded from the analysis (n = 4).

### Stroke significantly increases hippocampal neurogenesis

To quantify neurogenesis in adult DG following stroke, we analyzed the number of EdU positive cells which coexpressed the neuronal marker NeuN using confocal microscopy (Fig 1). The thymidine analogue EdU is incorporated by dividing precursor cells and remains immunohistochemically detectable following their differentiation into neurons (Fig 1A). Quantification of EdU+/NeuN+ cells revealed a significantly higher number of new neurons at day 49 following stroke versus control (1.5-fold, p<0,002 versus Sham-ST, Fig 1B). No significant difference was observed between the ipsi- and contralateral side in stroke or control animals.

In a second set of experiments, we used running to stimulate neurogenesis and carried out a similar quantification of EdU+/NeuN+ coexpression. Therefore, animals were housed in cages with free access to running wheels. The number of EdU+/NeuN+ cells was significantly increased in both running groups compared with animals housed under standard conditions (Fig 1B). Quantification yielded 1.32-fold increase in MCAO-RU (p<0,05 versus MCAO-ST) and 1.89-fold increase in sham operated runners (p<0,001 versus Sham-ST, Fig 1B).

### Stroke impairs spatial learning in Morris water maze

Next, we related our morphological results to behavioral data. To assess spatial memory following stroke, we trained our animals using a reference memory version of the MWM task with 6 trials per day for 5 days and a new platform position (reversal) on the beginning of day 4 [11]. The experimental set up of MWM included a high number of complex visual landmarks around the pool (e.g. different photographs with landscapes) and no curtain around the pool. Consequently mice were required to apply learning strategies that are more dependent on hippocampal neurogenesis [13].

In the first step, we analyzed the basic parameter of MWM comprising latencies (“time to find the platform”) and distance (“path lengths”) that mice needed to navigate to the platform. We found that following stroke mice generally needed more time to locate the platform. The longer latencies were detected throughout all five days during the acquisition and reversal period in stroke animals. The most prominent difference was observed at day 5 (26.73 sec) after goal reversal in MCAO-ST versus Sham-ST. The comparison of latencies between MCAO-ST and Sham-ST over 5 days reached statistical significance (p = 0.026, F6.607, ANOVA with multiple measures, Fig 2A). The length of the swim path in stroke animals was also significantly increased in MCAO-ST versus Sham-ST (p = 0.042, F5.318). Notably, the swim speed was not different in both groups (p = 0.904, F0.015, ANOVA with multiple measures).

Considering the latencies in both running groups, running did not improve the performance but even induced a more pronounced difference between stroke and control animals (Fig 2B, MCAO-RU vs Sham-RU, p = 0.010, F9.733).

In general, post hoc analysis yielded consistently shorter distances and latencies in all animals on day 3 compared to day 1 indicating a general successful task acquisition.

Comparing both controls, Sham-ST versus Sham-RU, we found no difference in the latencies (p = 0.492, F0.499, ANOVA using multiple measures). A similar result was obtained by comparing the latencies in both stroke groups, MCAO-ST versus MCAO-RU (p = 0.239, F1.593).

In summary, analysis of latencies and distance indicate that stroke animals show an impairment of spatial memory in the MWM despite the animals having a higher number of new neurons in the dentate gyrus.

In the next step, we investigated the quality of spatial learning. First, we plotted the probability of presence in the circular water maze at day 4 and could visualise a slower reversal learning in stroke animals (Heatmaps, Fig 2B). Second, we analyzed both the efficacy of the chosen search strategies and also whether the animals used more hippocampus-dependent or -independent strategies to locate the platform (Fig 3). The classification of search strategies was performed by using a mat lab algorithm as reported previously [11]. Statistical analysis employing generalized estimation equation revealed that in the acquisition phase, MCAO-ST used spatial strategies such as direct swimming, focal search, and the direct search significantly less frequently compared to the control (Fig 3). The estimated odds ratios yielded a >3-fold higher relative probability for controls (Sham-ST) to use spatial navigation from D1 to D3 (OR 7.62, p = 0.008 on D1, OR 3.15, p = 0.067 on D2. OR 3.82, p = 0.034 on D3, summarized in Fig 4). Analyzing all three hippocampus-dependent strategies following reversal of the platform, we found a robust trend showing that Sham-ST also used more specific spatial navigation versus MCAO-ST, however no significant difference was detected (OR 2.55, p = 0.11 on D4. OR 3.12, p = 0.13 on D5, Fig 4). On considering single strategies, we observed that direct swimming as the most efficient navigation to the platform was only employed by Sham-ST and not by MCAO-ST mice on days 4 and 5 indicating a important difference between both groups (Fig 3B). Taken together, analysis of single strategies unveils that stroke particularly decreases the application of hippocampus-dependent strategies in MWM.

Next we analyzed whether running as strong neurogenic stimulus might affect spatial learning in MCAO or sham-operated animals. We found that mice living in cages with free access to running wheels following MCAO still revealed an impaired memory performance although they experienced a further increase of new neurons in the DG. The latencies in the MWM between MCAO-RU and MCAO-ST were not significantly different from D1 to D5 (p = 0.239, F 1.593, Fig 2). The sham operated runners (Sham-RU) also needed almost identical times to localize the platform during acquisition and reversal similar to sham animals housed under standard conditions (Sham-ST, p = 0.492, F 0.499, Fig 2). Hence, running revealed no significant effect on the latencies in both control and stroke animals.

Analyzing the strategies in both running groups, animals with stroke (MCAO-RU) used a less efficient navigation strategy to find the platform. Hippocampal-dependent strategies were employed more frequently by the Sham-RU. The odds ratios calculated by general equation estimaton were constantly > 1 but significance was reached only at day 5 (OR 2.37, p = 0.419 on D1, OR 1.59, p = 0.289 on D2. OR 1.52, p = 0.532 on D3, OR 1.65, p = 0.437 on D4. OR 3.32, p = 0.042 on D5). The MCAO-RU rarely employed the efficient hippocampus-dependent strategies “direct swimming and focal search” particularly after goal reversal (Fig 3B). Here, focal search was used with a 2.96-fold higher probability by Sham-RU (p = 0.017).Similarly direct swimming was applied at 4.95-fold (OR) but the difference was not statistically significant (p = 0.118). However, the number of totally applied spatial strategies in the runnings groups compared to standard housing is more alike mainly during the acquisition phase (Fig 4), This finding indicates a beneficial effect of running on spatial memory following ischemia.

Thus, running does not improve the performance of MWM in general but promotes the application of spatial strategies in the acquisitions phase following ischemic insults.

### Impaired spatial memory performance correlates with aberrant hippocampal neurogenesis

In another set of experiments we addressed the question of whether the newly generated neurons reveal an aberrant morphology and location which is associated with malfunction of the hippocampal network [16,17]. In the intact brain, the mature GC reveal a stereotypic bipolar morphology with apical dendrites and an basal axon forming the mossy fiber tracts mainly in the hilar region [18].

**Fig 5:**
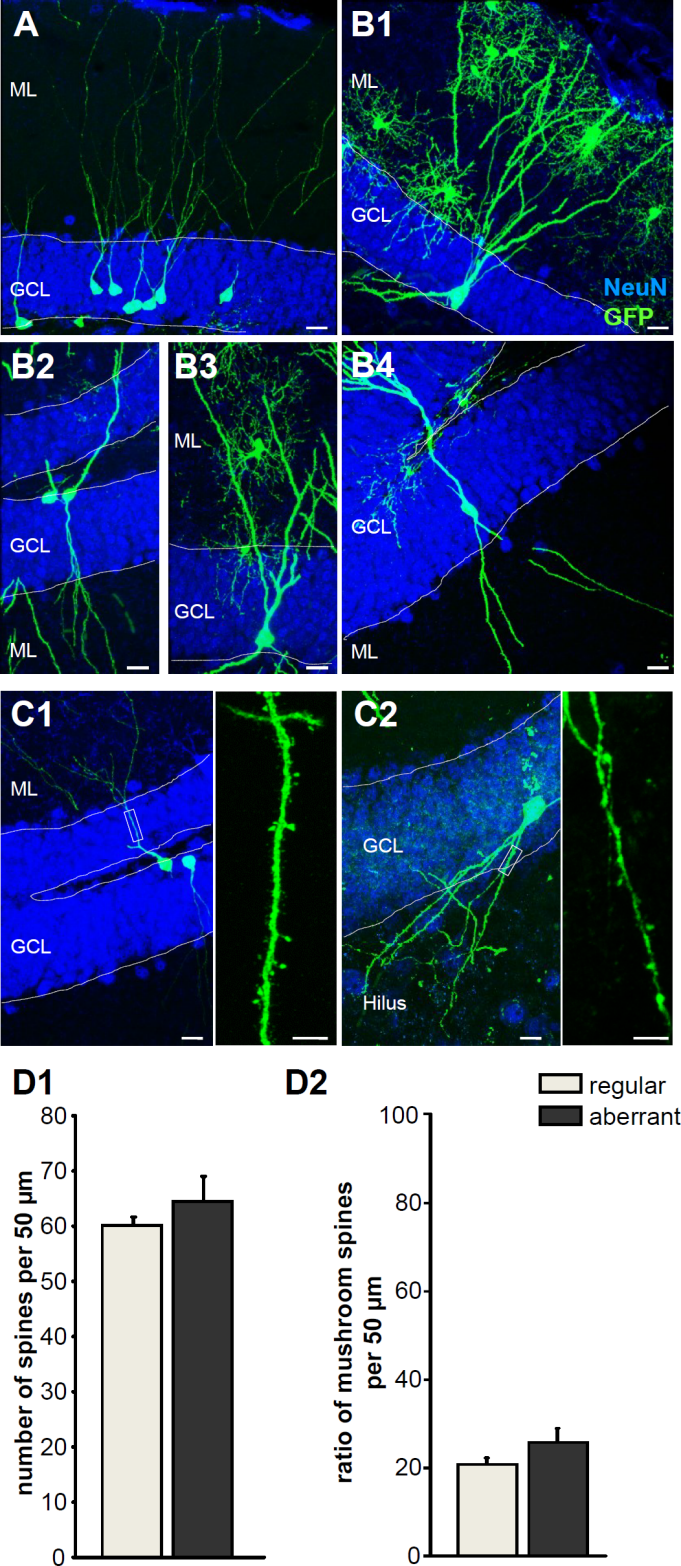
Stroke induces aberrant neurogenesis. **A,** Morphology of regular (“non-aberrant”) GFP-positive neurons after retroviral tracing in sham operated control. **B1-4,** Distinct examples of aberrant neurons following stroke expressing additional basal dendrites toward the hilus (bipolar cells). **C1,** Regular granule cell with characteristic dendritic tree spanning the granule cell layer. Right panel: higher magnification of one dendrite with spines. **C2**, Aberrant granule cell extending dendrites towards hilus. Right panel: higher magnification of one dendrite with spines. **D1-D2**, Quantification of spines in aberrant and regular granule cells (D1, total number of spines. D2, percentage of mushroom spines). Scale bars: 10μm (C1, C2 left) and 5μm (C1, C2 right).

Using retroviral labeling, we detected a significant number of new GC with atypical morphology and dendritic branching following ischemia. In particular, we observed GC displaying an additional dendritic tree extending from the basal pole towards the hilus (Fig 5) which is considered an immature feature of GC (MCAO-ST, n = 5/162 (3,09%), MCAO-RU, n = 8/91 (8,79%) Interestingly, running increased the number of neurons with aberrant dendritic branching poststroke (Fig 5) and we additionally oberserved singular ectopic granule cells in MCAO-RU. In the controls, Sham-ST and Sham-RU, we found only two cells with atypical morphology (n = 2/178 (1,12%). Taken together, ischemia increases the number of new born neurons but promotes aberrant dendritic branching indicating maladaptive plasticity in the DG.

Finally, we analyzed whether the aberrant neurons express spines as postsynaptic structures indicating network integration. Mushroom-shaped spines are considered mature whilst thin spines as immature. Quantification of spines by confocal microscopy revealed an almost similar number of mushroom spines on aberrant and regular granule cells (Fig 5D1). The ratio of mushroom and thin spines was similar in both granule cell types (Fig 5D2). In summary, the expression of mature mushroom spines by aberrant cells suggest that they are synaptically integrated and can influence the hippocampal network.

## Discussion

Stroke significantly stimulates neurogenesis in the adult dentate gyrus, although the functional role of this postlesional response is mostly unclear. Notably, higher numbers of new neurons do not reliably correlate with better outcome poststroke [9]. Recent findings even suggest that a significant portion of new neurons become aberrantly integrated [10]. Here, we asked the question whether stroke in fact increases the number of new granule cells but thereby also promotes aberrant neurogenesis which subsequently impairs specific learning capabilities that involve newly generated granule cells in the hippocampus.

Using a modified version of the Morris water maze [13] and distinct labeling methods of new neurons, we demonstrate that following middle cerebry artery occlusion, mice revealed a significant impairment relating to the hippocampus-dependent memory tasks. Further, a detailed morphological analysis yielded aberrant neurogenesis in the DG after stroke. Thereby, the total number of newborn neurons increased following MCAO.

In the physiological state, newborn GC in the adult DG undergo a multi-stage maturation process before becoming fully integrated in the pre-existing neuronal circuitry. The neurite outgrowth follows an orderly spatiotemporal pattern. The immature neurons initially send out many short processes. One process from the basal cell pole elongates more rapidly to become the axon. The emergence of this axon extending to the CA3 region within 7–10 days coincides with the appearance of apical dendrites [18]. Basal dendrites are also formed a few days earlier but are merely a development feature of GC and are mostly retracted at day 7. After a period of maximal neurite and spine growth as well as enhanced synaptic plasticity, the new GC form relatively stable synaptic connections within four to six weeks. This process is strictly regulated and < 1% of new neurons reveal aberrant connections in the intact brain [4]. Different brain insults can significantly change the differentiation and integration of GC in the DG. In the rodent model of temporal lobe epilepsy, about 10% of GC display persistent hilar basal dendrites [4] forming aberrant circuitries which contribute to hyperexcitability in the hippocampus [19,20]. Besides aberrant dendritic branching, ectopic GC in the hilus can also be observed in the epileptic brain indicating an ectopic migration of some neurons [4]. Importantly, in epilepsy models, aberrant new neurons were regularly associated with a general increase of neurogenesis in DG [21,22].

In the ischemic brain, we previously demonstrated that increased neurogenesis was associated with aberrant dendritic connections and ectopic location of new GC in rats [10] although the functional impact of this observation remained unclear. In the present study, we now prove that aberrant neurogenesis also occurs in the murine hippocampus and is associated with poorer performance in hippocampal-dependent memory tasks following focal infarcts.

The number of viral-labelled aberrant neurons in our study is low which might question their functional impact and possibly limit the significance of our findings. Our approach of using a single stereotactic injection of retroviruses into the intrahippocampus allows labelling of only a small fraction of the cells. This is because, in order to be labelled, transfected cells need to be close to the injection site and be in a process of cell division. And because aberrant integration occurs in only 10–20% of newly born neurons in the lesioned brain, the probability of labeling an aberrant neuron is further reduced.

In epilepsy models in which neurogenesis is generally more enhanced in comparison to models using ischemic infarcts, viral labeling of newly generated cells has also been performed. In these models, the extent of aberrant neurogenesis was also low but had a significant impact on network function [23,24]. Myers et al. recently demonstrated that even a small population of aberrant granule cells (about 5%) caused a significant impairment of spatial memory by changing the backprojections from the CA3 region [25]. In summary, these studies conclusively indicate that even a low number of aberrant neurons severely affects hippocampal function.

The analysis of the functional impact of adult neurogenesis in behavioral experiments or cognitive tasks remains challenging. The water maze still represents the gold standard for testing hippocampal function in rodents. However, the application of standard protocol and the analysis of basic parameters of the MWM yielded conflicting data related to adult neurogenesis in the DG.

The newly generated neurons do not appear to be beneficial for hippocampal function per se, but rather contribute to highly specific functional aspects of spatial learning that become apparent only when certain task demands are encountered or when the spatial memory is stressed [13]. The new neurons particularly improve the ability to separate distinct complex visual contexts [5,26] and allow rapid adaptation to a new context. Here we employed a modified version of the MWM comprising complex visual landmarks and goal reversal at day 4, which challenges the hippocampal function. We additionally used read out parameters that are more specifically related to the functional contribution of adult neurogenesis [11]. Rapid adaption is particularly challenged after the platform position is changed on days 4 and 5 in MWM. We observed the major difference of latencies at day 5 and a complete lack of the precise search strategy “direct swim” in MCAO-ST in comparison to Sham-ST.

Running which is considered as a classic neurogenic stimulus, further increased the number of newly generated neurons but also enhanced aberrant neurogenesis poststroke. By increasing neurogenesis in the hippocampus, running apparently also raises the probability of aberrant integration and does not successfully counteract maladaptive hippocampal plasticity in the ischemic brain. The analysis of the latencies in MWM consistently revealed a significant difference between MCAO and Sham in both running groups, particularly after goal reversal (Fig 2B). However, comparing the strategies, the MCAO-RU animals used a similar level of spatial navigation (Fig 4B) as Sham-RU, particularly on days 1–3, indicating at least a modest benefit from the increased hippocampal neurogenesis.

Although running is associated with more new neurons in the hippocampus, we observed no significant improvement in MWM (comparing Sham-RU vs Sham-ST)in the sham operated groups, This finding can be explained by the fact that adult neurogenesis only becomes involved in spatial memory when the information processing capacity in the DG is sufficiently stressed [13] e.g. poststroke. Under more “physiological conditions” the sensitivity of our MWM might be not high enough to show minor differences between runners and non-runners in the sham operated groups with sample sizes of 7–8 animals per group.

Finally, stroke also induced differences in MWM strategies that are considered hippocampal independent (Fig 3) pointing to a stroke-induced broader disruption of hippocampal circuitry which goes beyond the effect of aberrant neurogenesis.

Although clinical obervations indicate that stroke survivors frequently develop mild to moderate cognitive deficits [2,27] related to the hippocampus, the etiology of this phenomenon is not yet understood. Our data provide a possible clue to the cellular mechanisms that might contribute to memory impairment poststroke.

## Conclusions

Taken together our results demonstrate that ischemic insults cause hippocampal-dependent memory deficits which are associated with aberrant neurogenesis in the dentate gyrus indicating an ischemia-induced maladaptive plasticity in remote brain areas. More studies are needed to clarify whether tailored rehabilitative and therapeutic approaches might counteract aberrant neurogenesis and improve hippocampal-dependent memory performance.

## Supporting information

S1 Fig Illustration of experimental design and stroke-induced lesion. **A,** Adult mice were allocated to four experimental groups: MCAO or Sham operation with standard housing or free access to running wheels, respectively. Proliferation marker EdU was injected for 2 weeks starting 3 days postsurgery. GFP-retrovirus was injected 4 days after surgery. Spatial learning was assessed in the Morris water maze (MWM) at days 42 to 46. **B,** Representative images of coronal brain slices, control and MCAO, red frame indicates ischemic insult of the left hemisphere.
(PDF)

S1 Data Supporting information. Data of cell counts, behavioural experiments and analysis of neuron morphology.
(XLSX)
