# Supplementary material for: Adult hippocampal neurogenesis poststroke: More new granule cells but aberrant morphology and impaired spatial memory
Source: PLoS One. 2017 Sep 14;12(9):e0183463. doi: 10.1371/journal.pone.0183463 (PMC5598932; doi:10.1371/journal.pone.0183463)
Supplement: S1 Fig — A, Adult mice were allocated to four experimental groups: MCAO or Sham operation with standard housing or free access to running wheels, respectively. Proliferation marker EdU was injected for 2 weeks starting 3 days postsurgery. GFP-retrovirus was injected 4 days after surgery. Spatial learning was assessed in the Morris water maze (MWM) at days 42 to 46. B, Representative images of coronal brain slices, control and MCAO, red frame indicates ischemic insult of the left hemisphere. (PDF) [file pone.0183463.s001.pdf]

Supplemental Figure 1

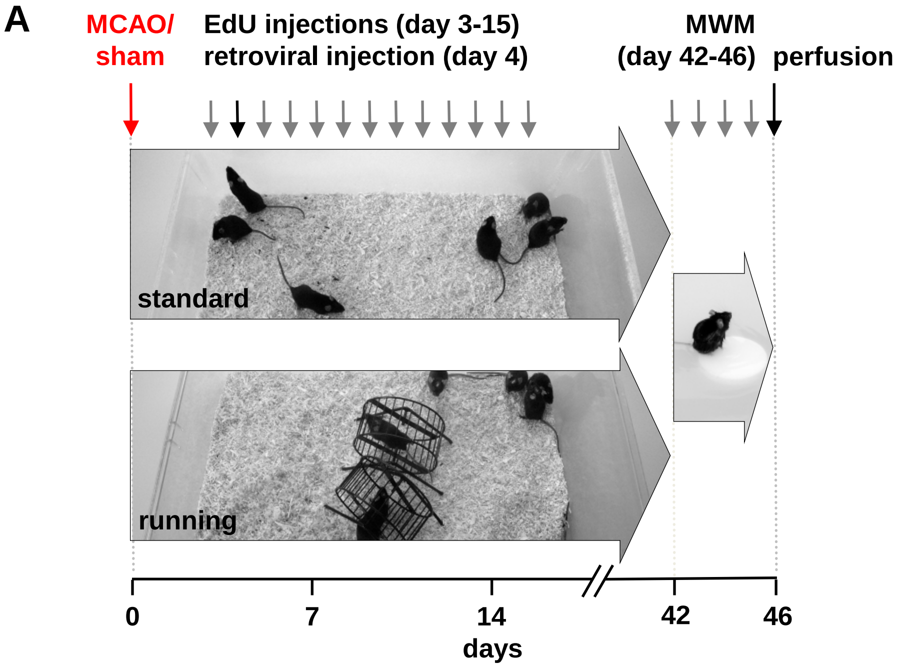

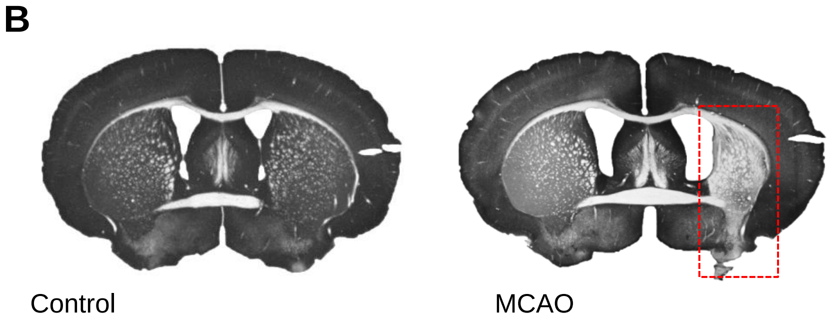

Supplemental Figure 1: A, Schematic illustration of experimental design. Adult mice were allocated to four experimental groups: MCAO or Sham operation with standard housing or free access to running wheels, respectively. Proliferation marker EdU was injected for 2 weeks starting 3 days postsurgery. GFP-retrovirus was injected 4 days after surgery. Spatial learning was assessed in the Morris water maze (MWM) at days 42 to 46. **B,** Representative images of coronal brain slices, control and MCAO, red frame indicates ischemic insult of the left hemisphere.
